# Differential Expression of CD3, TNF-α, and VEGF Induced by Olanzapine on the Spleen of Adult Male Albino Rats and the Possible Protective Role of Vitamin C

**DOI:** 10.3390/biomedicines7020039

**Published:** 2019-05-23

**Authors:** Sahar Youssef, Marwa Salah

**Affiliations:** 1 Anatomy Department, Faculty of Medicine for Girls, Al-Azhar University, Cairo 11754, Egypt; 2 Zoology Department, Faculty of Science, Beni-Suef University, Beni-Suef 62514, Egypt; Marwa_Salah78@yahoo.com

**Keywords:** CD3, olanzapine, spleen, TNF-α, VEGF, vitamin C

## Abstract

Olanzapine is an antipsychotic drug effective in the treatment of stress-associated psychiatric illnesses, but its effect on the spleen remains unclear. Vitamin C is essential for the optimum function of the immune system. We aim to investigate the effect of Olanzapine on spleen structures and to assess the protective effect of vitamin C. Forty adult male albino rats were divided into four groups: group (I), a control; group (II), rats were given vitamin C at 40 mg/kg body weight; group (III), rats were given Olanzapine at 2 mg/kg body weight; and group (IV), rats were given vitamin C and Olanzapine at the same dose of group (II) and group (III) for one month. The hematoxylin and eosin (H&E) of the olanzapine treated group showed focal areas of cellular depletion and a decrease in the size of the white pulp. The red pulp was expanded and showed marked congestion and dilatation of blood sinusoids. Cluster of differentiation 3 (CD3) was significantly reduced, however both tumor necrosis factor alpha (TNF-α), and vascular endothelial growth factor (VEGF) were significantly higher. The administration of vitamin C repaired structural and immunohistochemical changes via increased CD3 and decreased TNF-α and VEGF. Therefore, the oxidative and the inflammatory pathways may be the possible mechanisms underlying olanzapine immunotoxicity. Vitamin C exerted immune modulator and antioxidant effects against olanzapine.

## 1. Introduction

Olanzapine is one of the antipsychotic drugs used broadly in the treatment of psychotic conditions. It is also used as monotherapy or in combination with antidepressants to treat depressive illnesses [1]. The human therapeutic dose is 10–20 mg/day [2]. Few side effects have been reported for Olanzapine, such as severe pruritic skin eruption [3]. Some investigators observed that Olanzapine induced hepatic toxicity in few cases [4]. Olanzapine induces weight gain, insulin resistance, hyperglycemia, and dyslipidemia [5]. Adverse effects due to olanzapine appear to be dose-dependent [6]. Few studies on Olanzapine-induced alterations in the architecture of the spleen have been reported. The effects of lactation exposure to Olanzapine (4, 8, and 10 mg/kg) on hematology, spleen, and thymus of mice neonates were investigated. Olanzapine has suppressed the lymphoid organs, such as the thymus and spleen, and decreased the number of follicles of the spleen [7].

Olanzapine was studied in vitro and induced cytotoxic effects on cultured lymphocytes. Some researchers suggested that the cytotoxicity of this drug is associated with oxidative stress. Indeed, oxidative stress and the associated production of reactive oxygen species (ROS) have been integrated and involved in the clinical harmful effects of antipsychotics [8]. Numerous studies have reported that the generation of ROS, including superoxide radicals, hydrogen peroxide, hydroxyl radicals, and lipid peroxides, produced damage to cellular components, such as protein, DNA, and membrane lipids. Mitochondrial ROS overproduction and alterations in mitochondrial redox homeostasis are involved in many neurological and somatic disorders [9].

The immune system is one of the crucial systems in the body which might be affected by numerous factors and various pharmaceutical drugs, leading to immune stimulation or immunosuppression [10]. The spleen is the largest immune organ in the body and plays an important role in immune defense due to the presence of lymphocytes and macrophages. It acts as the main source to produce immunocytokines, as well as tumour necrosis factor (TNF-α), which mediate the immune response in the immune system [11].

Vitamin C or ascorbic acid is a natural antioxidant that inhibits oxidative impairment in body tissues [12]. It is vital for cellular growth and differentiation. Vitamin C is essential for the optimum function of the immune system [13]. There are suggestions of a biokinetic association between ascorbic acid dose and immune cell concentration that emphasizes its specific function in the immune response [14]. The biological purpose of ascorbic acid is based on its capability to provide decreasing various biochemical reactions, because vitamin C can reduce most physiologically-relevant reactive oxygen species. The antioxidant role of ascorbic acid contributes to the protection of cells from the harmful effects of exogenous or endogenous ROS and reactive nitrogen species (RNS), that can form either during toxicant exposure or during inflammatory reactions in a host. Crucially, vitamin C was shown to improve components of the human immune system, such as natural killer cell actions, lymphocytes proliferation, and chemotaxis [15]. 

The spleen was selected for the current study to assess the effect of Olanzapine, as some investigators [16] suggested that the spleen is one of the most important parts of the immune system, as it has a higher sensitivity to low doses of chemicals than the other organs. 

A definitive link between the action of Olanzapine and cluster of differentiation 3 (CD3), tumor necrosis factor alpha (TNF-α), and vascular endothelial growth factor (VEGF) is yet to be elucidated. Based on earlier investigations, the objective of the current study was to explore immunomodulatory changes induced by Olanzapine in the spleen of adult male albino rats and the possibility of protection by co-administration with vitamin C.

## 2. Material and Methods

### 2.1. Animals and Housing

Forty adult male albino rats weighing approximately 200–220 g were purchased from the animal house of the Faculty of Medicine, Assiut University, Egypt. Rats were preserved under harmless sterile laboratory conditions, a 12 h dark and 12 h light cycle. Rats treated according to the guidelines of the Animal House of Assiut University, where standard commercial pellets for feeding, water ad libitum were performed. All animal procedures were in accordance with the guidelines for the care and use of experimental animals by the Committee for the Purpose of Supervision of Experiments on Animals (CPCSEA) and according to the National Institute of Health protocol.

### 2.2. Chemicals

Olanzapine (Zyprexa TM) was presented in the form of 10 mg tablets, obtained from Eli Lilly Company, Indianapolis, IN, USA. Vitamin C was presented in the form of 500 mg tablets obtained from Seif pharmacy, Cairo, Egypt.

### 2.3. Experimental Design

Rats were randomly distributed into four groups (*n* = 10/group): group (I) (control group) rats received 1 mL distilled water daily (Olanzapine vehicle); group (II) (Vitamin C group) rats received 40 mg/kg body weight [17]; group (III) (Olanzapine group) rats received 2 mg/kg body weight of Olanzapine, which is equal to a human dose [2]; group (IV) (Olanzapine and vitamin C group) rats received the same dose of Olanzapine and vitamin C for the same period as group (II) and group (III). The Olanzapine dose was calculated according to Paget and Branes [18]. Olanzapine and vitamin C were given orally and daily for four weeks.

### 2.4. Histological Study

After one month, rats from different studied groups were sacrificed and their spleens were collected and prepared for light microscopic studies. The spleen specimens were fixed, dehydrated, and cleared in xylene. Paraffin sections of 5 μm were cut, deparaffinized, and stained with hematoxylin and eosin (H&E) for histological analysis [19].

### 2.5. Immunohistochemistry

The immunohistochemical staining was achieved by a streptavidin-biotin-peroxidase method [20]. Sections of all studied groups were stained with rabbit monoclonal antibody (SP7) (abcam, 16669, Cambridge, UK) against CD3 at dilution 1:100, rabbit polyclonal antibody against TNF-α (abcam, ab6671, Cambridge, UK) at 1:200, and the rabbit polyclonal antibody against VEGF at 1:500 (Gene Tex, USA). The paraffin sections with 3–5 μm thickness were immersed with xylol to eliminate paraffin. Dehydration was done in descending grades of alcohol and then washed twice with distilled water for five minutes. Endogenous peroxidases were blocked by usage 5% hydrogen peroxidase for ten minutes. Antigen retrieval and antibody dilution were completed according to manufacturers’ instructions. Sections were incubated with the primary antibody for 60 min at 25 °C, followed by washing, then incubated for 30 min with the biotinylated secondary antibody. The 3,3′-diaminobenzidine (Dako, Glostrup, Denmark) at pH 7.0 for three minutes was used as a chromogen. The slides were counterstained with Mayer’s hematoxylin, dehydrated, and mounted with Dibutylphthalate Polystyrene Xylene (DPX). The negative control section from each group was incubated with Phosphate Buffered Saline (PBS) without the addition of the primary antibody.

### 2.6. Morphometric Study

Morphometric measurements were obtained using computer-based image analysis software (Leica QWin 500, Cambridge, UK) to measure the area percentage of the red pulp, white pulp, TNF-α, and VEGF immunoreactivity. Ten randomly selected low power fields from each slide were analyzed and expressed as a mean area percentage of the total area. Immunopositive optical density for CD 3 was also assessed. The optical density was estimated randomly in the periarterial lymphatic sheath of spleens from 10 randomly selected non-overlapping fields. CD3 morphometric measurements were taken at a total magnification of 400×. 

### 2.7. Statistical Analysis

The data were subjected to statistical analysis using one-way analysis of variance performed with (ANOVA). The student’s *t*-test was used for comparison between groups. The significance of the data was performed by the *p* value. *p* values less than 0.05 were considered significant. Data were expressed as mean ± standard deviation (SD). 

## 3. Results

### 3.1. H&E Results

**Figure 1:**
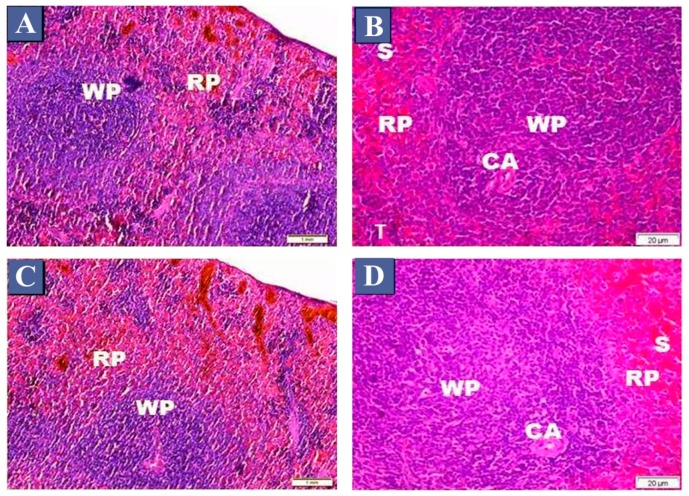
Photomicrographs of haematoxylin and eosin (H&E) stained sections of the spleen from the control group and vitamin C group. (**A**,**B**) the control group, showing the normal arrangement of white pulp (WP) and red pulp (RP). White pulp (WP) contains the central arteriole (CA), surrounded by numerous lymphocytes. Note the darkly stained nuclei of the lymphocytes. Part of the red pulp (RP) with splenic cords have lymphocytes, blood sinusoids (S) and trabeculae (T). (**C**,**D**) The vitamin C group, showing the normal architecture of white pulp (WP), central arteriole (CA), red pulp (RP) and blood sinusoids (S). (**A**,**C**) H&E, ×100, (**B**,**D**) H&E, ×400.

Examination of H&E-stained sections of the control group showed that the spleen consists of two major components, white pulp and red pulp (Figure 1A,B). The white pulp consists of follicles with a peripherally-located central arteriole, surrounded by a sheath of lymphocytes (Figure 1B). The red pulp consists of branching and anastomosing splenic cords with blood sinusoids in between (Figure 1B). Examination of H&E-stained sections of the vitamin C-treated group (II) showed that they consisted of the normal architecture of white pulp with a sheath of numerous lymphocytes surrounding the central arteriole (Figure 1C,D). The red pulp was composed of splenic cords and blood sinusoids (Figure 1D). 

**Figure 2:**
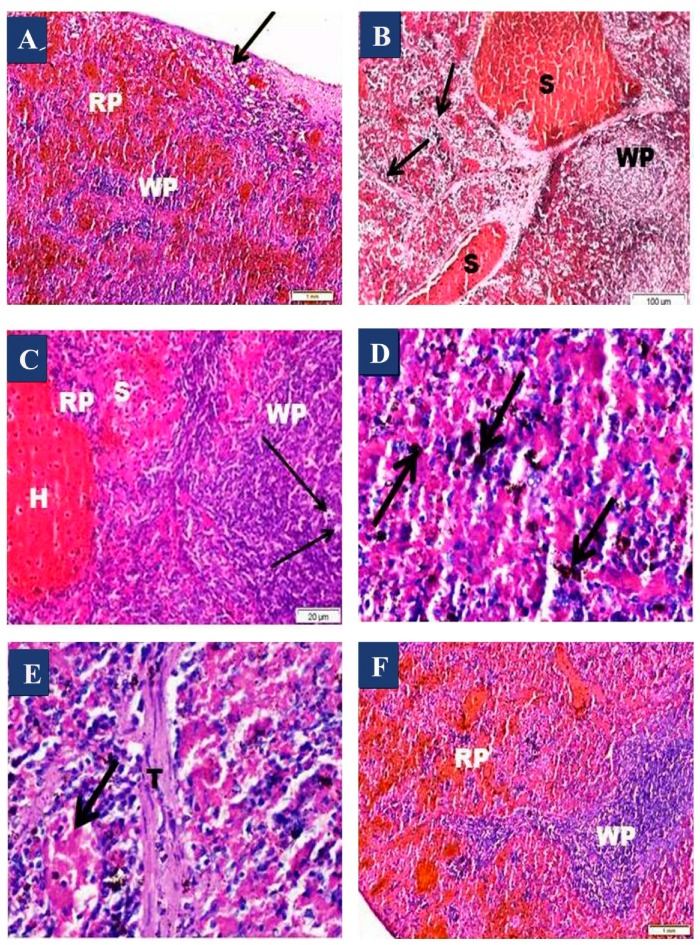
Photomicrographs of haematoxylin and eosin stained sections of the spleen from group (III) and group (IV). (**A**) Olanzapine-treated group showing loss of normal architecture and highly shrunken white pulp (WP) and broadened red pulp (RP). Note the massive area of vacuolations (arrow). (**B**) The Olanzapine-treated group showing congested dilated splenic sinuses (S) in the red pulp. Many cells in the white pulp (WP) appear vacuolated. Note, thick fibrous trabeculae is observed (arrows). (**C**) The Olanzapine-treated group showing some cells that appeared vacuolated with fragmented nuclei (arrows) in white pulp (WP), massive hemorrhage (H) in the red pulp (RP), and dilated sinusoids (S). (**D**) Olanzapine group showing hemosiderin laden cells (arrows). (**E**) Olanzapine group showing thickened trabeculae (T) and markedly dilated congested splenic sinus (arrow). (**F**) Vitamin C and olanzapine group showing nearly normal appearance of the splenic architecture of white pulp (WP) and red pulp (RP). (**A**,**B**,**F**) H&E, ×100; (**C**–**E**) H&E, ×400.

An examination of H&E-stained sections of the Olanzapine-treated group (III) showed atrophied white pulp and massive vacuolations (Figure 2A). The white pulp revealed focal areas of cellular depletions (Figure 2B). Some cells appeared vacuolated, with fragmented nuclei in white pulp (Figure 2C). The red pulp was expanded and showed marked congestion of its dilated blood sinusoids and a massive hemorrhage (Figure 2B,E). Hemosiderin laden macrophages (Figure 2D) and thickening of splenic trabeculae were observed (Figure 2E). Spleen sections of the Olanzapine- and vitamin C-treated group (IV) showed an apparent increase in the size of the lymphatic follicles in comparison to group (III). Preserved architecture of white pulp and red pulp with slightly dilated blood sinusoids was observed (Figure 2F).

**Figure 3:**
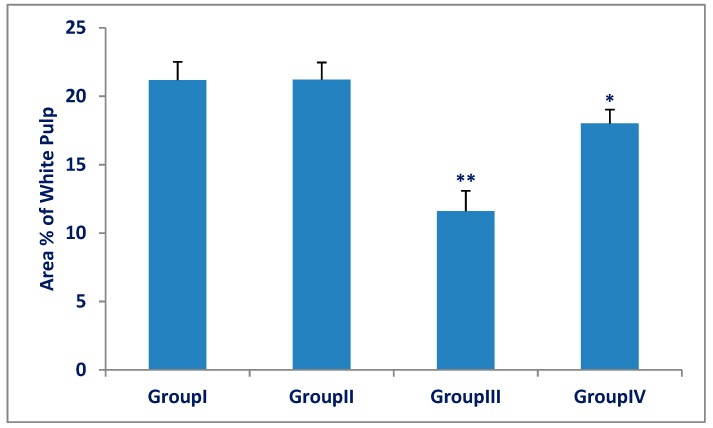
Area percentage of the white pulp of the spleen in group (III) (Olanzapine group) was significantly decreased as compared to group (I) (control group). A significant increase in group (IV) (Olanzapine and vitamin C) as compared to group (III).The differences in area percentage of white pulp between group (I) and group (II) were not significant (*p* > 0.05). Results are expressed as mean ± SD and the level of significance was set for *p*-values less than 0.05. **: significant decrease, group (III) versus group (I) (*p* < 0.001). *: significant increase, group (IV) versus group (III) (*p* < 0.001).

**Figure 4:**
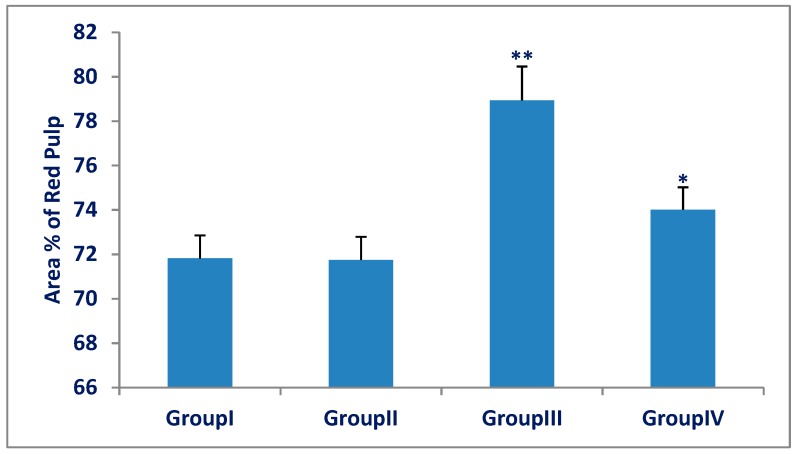
Area percentage of red pulp of spleens in group (III) (Olanzapine group) was significantly increased as compared to group (I) (control group). Group (IV) (Olanzapine and vitamin C) was significantly decreased as compared to group (III). The differences in area percentage of red pulp between the control group (I) and Vitamin C group (II) were not significant (*p* > 0.05). Results are expressed as mean ± SD and the level of significance was set for *p*-values less than 0.05. **: significant increase, group (III) versus group (I) (*p* < 0.001). *: significant decrease, group (IV) versus group (III) (*p* < 0.001).

The white and red pulp area percentage for all groups is represented in Figure 3 and Figure 4. There was a significant decrease (*p* < 0.001) in white pulp area percentage in the Olanzapine-treated group (III) compared with the control group (I). There was a significant increase (*p* < 0.001) in the white pulp in the Olanzapine- and vitamin-C treated group (IV) compared with that of Olanzapine-treated group (III). There was a significant increase (*p* < 0.001) in red pulp area percentage in the Olanzapine-treated group (III) compared with the control group (I). There was a significant decrease (*p* < 0.001) in the red pulp area percentage in the Olanzapine- and vitamin C-treated group (IV) compared with that of the Olanzapine-treated group (III).

### 3.2. CD3 Immunohistochemistry and Morphometric Results

**Figure 5:**
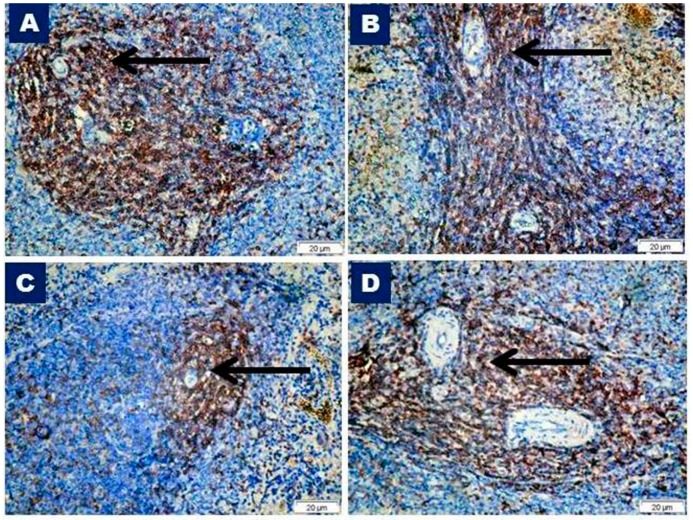
Photomicrographs of immunohistochemically-stained sections of the spleen with anti- cluster of differentiation 3 (CD3) from different studied groups. (**A**) Control group (I), (**B**) Vitamin C group (II) showing strong positive T-lymphocytes in periarteriolar areas (arrows). (**C**) Olanzapine treated group (III) showing a decreased CD3 positive reaction in the periarterial lymphatic sheath (arrow). (**D**) Vitamin C and Olanzapine treated group (IV) showing an increased CD3 positive reaction in the periarteriolar areas (arrow). CD3, ×400

The CD3 antibody recognized T cells by the cell membrane and cytoplasmic reactions. The immunohistochemical staining of CD3 cells in the spleen of control sections showed strong positive cells in the periarterial lymphatic sheath (Figure 5A). Strong staining in the vitamin C-treated group was detected (Figure 5B). There was a significant reduction in the CD3 immunopositive reaction in the periarterial lymphatic sheath in the Olanzapine-treated group (Figure 5C). The CD3 positive reaction was significantly increased in the Olanzapine- and vitamin C-treated group (Figure 5D), as compared with that of the Olanzapine-treated group (Figure 5C), but less than that of the control group (Figure 5A).

**Figure 6:**
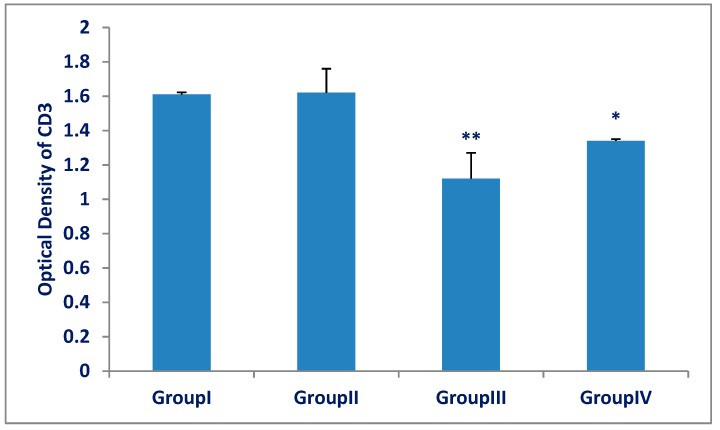
The optical density of CD3 in the spleens of group (III) (Olanzapine-treated group) was significantly decreased as compared to group (I) (control group). A significant increase in group (IV) (Olanzapine and vitamin C) as compared to group (III). The differences in optical density between group (I) and group (II) were not significant (*p* > 0.05). Results are expressed as mean ± SD and the level of significance was set for *p*-values less than 0.05. **: significant decrease, group (III) versus group (I) (*p* < 0.001). *: significant increase, group (IV) versus group (III) (*p* < 0.001).

The mean optical density of CD3 expression for all groups is represented in Figure 6. There was a significant decrease (*p* < 0.001) in CD3 expression in the Olanzapine-treated group (III) compared with that of the control group (I). There was a significant increase (*p* < 0.001) in CD3 expression in the Olanzapine- and vitamin C-treated group (IV) compared with that of the Olanzapine-treated group (III).

### 3.3. TNF-α and VEGF Immunohistochemistry and Morphometric Results

**Figure 7:**
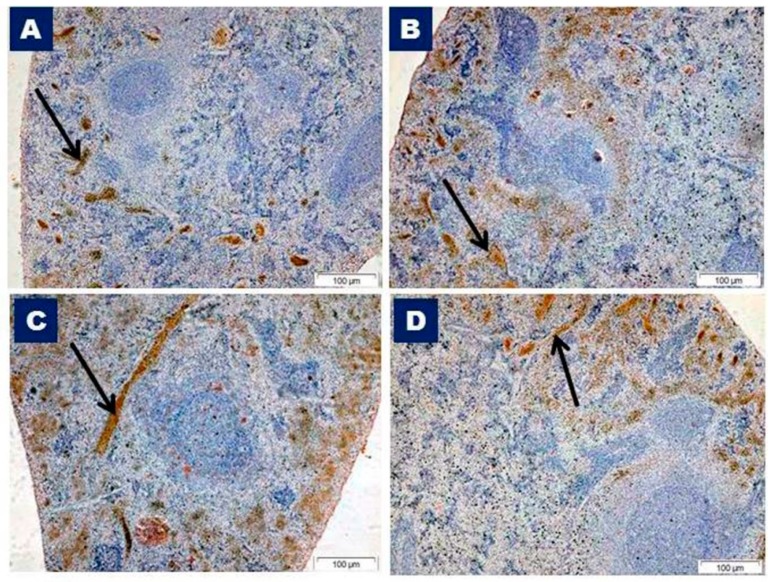
Photomicrographs of immunohistochemically-stained sections of the spleen with anti- tumor necrosis factor alpha (TNF-α) from different studied groups. (**A**) Splenic section from the control group showing negatively stained white pulp, with positive brown staining in the red pulp (arrow). (**B**) Splenic section from the vitamin C group showing a positive brown reaction in the red pulp (arrow) and no immunostain in the white pulp. (**C**) Splenic section of the Olanzapine-treated group showing increased immunoreactivity in the red pulp (arrow) with negatively stained white pulp. (**D**) Splenic section from the Olanzapine and vitamin C group showing moderate staining in the red pulp (arrow) and negatively stained white pulp. TNF-α immunostaining, ×100.

Immunostained control sections of TNF-α showed a positive cytoplasmic reaction within the cells of the red pulp, with negatively stained white pulp (Figure 7A). The TNF-α immunopositive reaction of vitamin C sections revealed a similar staining pattern to the control group (Figure 7B). Increased immunoreactivity in the red pulp and no staining in the white pulp of the Olanzapine-treated group sections were observed (Figure 7C). The Olanzapine-and vitamin C-treated group revealed decreased immunostaining in the red pulp (Figure 7D) than that of the Olanzapine-treated group, but more than that of the control group (Figure 7A).

**Figure 8:**
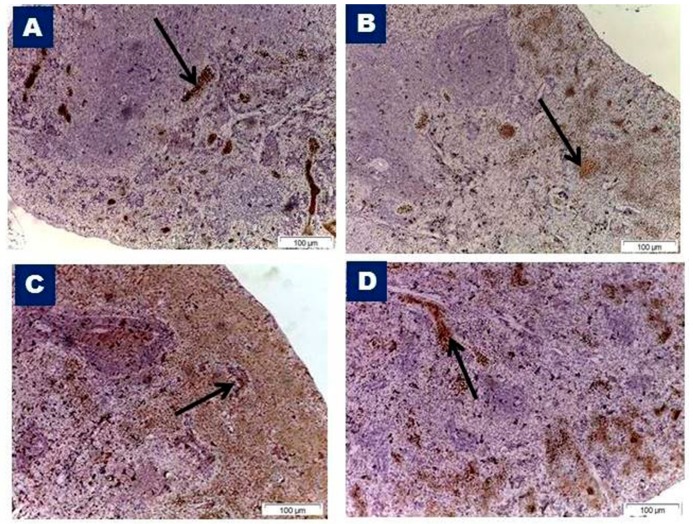
Photomicrographs of immunohistochemically-stained sections of the spleen with anti- vascular endothelial growth factor (VEGF) from different studied groups. (**A**) Control group, (**B**) vitamin C group showing positive brown immunoreactivity (arrows) within the red pulp, with negatively stained white pulp. (**C**) Olanzapine-treated group showing a marked increase in immunostaining within the red pulp (arrow) and negatively stained white pulp. Note, strong brown cytoplasmic staining within endothelial cells lining the venous sinuses. (**D**) Olanzapine and vitamin C group showing moderate immunostaining within the red pulp in the endothelial cells of venous sinuses (arrow) but no immunoreactivity within the white pulp. VEGF immunostaining, ×100.

VEGF-immunostained control sections showed a positive reaction in the endothelial cells lining the sinusoids of the red pulp (Figure 8A). The vitamin C group showed a similar immunopositive reaction (Figure 8B) to the control group. An examination of the Olanzapine-treated group sections revealed a marked increase in immunoreactivity identified as brown cytoplasmic staining within the endothelial cells lining the venous sinuses (Figure 8C). The Olanzapine- and vitamin C-treated group sections revealed decreased immunopositivity in the endothelial cells of venous sinuses (Figure 8D) as compared to olanzapine.

**Figure 9:**
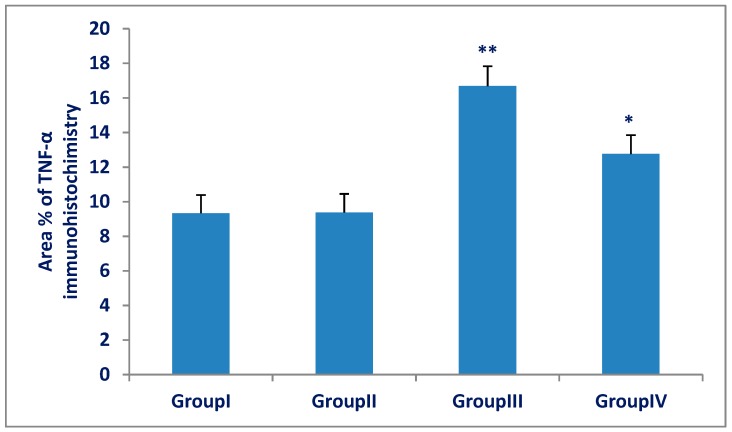
Area percentage of TNF-α of spleen in group (III) (Olanzapine-treated group) was significantly increased compared to group (I) (control group). A significant decrease in group (IV) (Olanzapine and vitamin C) as compared to group (III). The differences in area percentage of TNF-α between group (I) and group (II) were not significant (*p* > 0.05). Results are expressed as mean ± SD and the level of significance was set for *p*-values less than 0.05. **: significant increase, group (III) versus group (I) (*p* < 0.001). *: significant decrease, group (IV) versus group (III) (*p* < 0.001).

**Figure 10:**
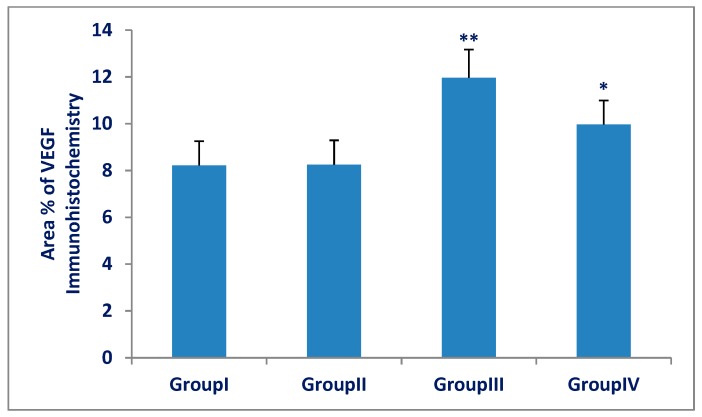
Area percentage of VEGF of spleens in group (III) (Olanzapine-treated group) was significantly increased compared to group (I) (control group). The Olanzapine and vitamin C group (IV) was significantly decreased as compared to group (III). The differences in area percentage of VEGF between group (I) and group (II) were not significant (*p* > 0.05). Results are expressed as mean ± SD and the level of significance was set for *p*-values less than 0.05. **: significant increase, group (III) versus group (I) (*p* < 0.001). *: significant decrease, group (IV) versus group (III) (*p* < 0.001).

The mean area percentage of TNF-α and VEGF expression for all groups is represented in Figure 9 and Figure 10. There was a significant increase (*p* < 0.001) in TNF-α and VEGF expression in the Olanzapine-treated group (III) compared with that of the control group (I). There was a significant decrease (*p* < 0.001) in TNF-α and VEGF expression in the Olanzapine- and vitamin C-treated group (IV) compared with that of the Olanzapine-treated group (III), but more than that of the control group.

## 4. Discussion

The morphometric analysis of the spleen in the present study showed a decreased area percentage of white pulp in the Olanzapine-treated group, suggesting immune response alterations in these animals. The loss of white pulp and reduction in splenic lymphocytes may be via apoptosis, which was confirmed by the presence of fragmented nuclei in some cells. Some investigators suggested that white pulp atrophy is one of the most common findings following certain immunosuppressive drugs and are accompanied by the reduced capability of the animal’s immune system to produce antibodies [21]. 

Regarding the histological changes in the present study, the Olanzapine-treated rats showed focal areas of cellular depletion in the white pulp and large congested blood vessels. Oxidative stress may be the most serious component in the pathophysiology and toxicities related to antipsychotic treatments [22]. A study from a rat model proposed that alterations and cytotoxicity of olanzapine in the liver cells were started by an over-production of ROS, which led to mitochondrial collapse and lysosome membrane leakage. Furthermore, it resulted in decreased lipid peroxidation and glutathione depletion and finally cell lysis [23]. Some investigators reported that oxidative inflammatory mechanisms have been hypothesized to perform a role in the onset of metabolic adverse effects related to olanzapine administration [24]. Olanzapine treatment of macrophages elevated superoxide and ROS, such as hydrogen peroxide, these findings suggest that olanzapine may lead to mitochondrial dysfunction [25].

The effects of lactational exposure to olanzapine on hematology and lymphoid organs in mice neonates were studied [7]. They reported that follicular size, germinal centers, and size of splenocytes were decreased in both white and red pulp. The density of splenocytes in the white pulp, with darkly stained apoptotic lymphocytes, was observed. Moreover, the area of red pulp was increased, with an increased number of splenic sinuses and hematopoietic cells. They attributed these changes to corticosteronemia and hyperprolactinemia [7].

The vacuolar degeneration observed in the parenchyma of the spleens treated with Olanzapine was consistent with other researchers, who suggested that silver nanoparticles induced autophagosomes containing cellular debris, disintegrated materials, lipid droplets, and changed intracellular homeostasis [26,27]. 

Hemosiderin laden cells were observed in the red pulp of the Olanzapine-treated group. Splenic macrophages were activated to eradicate old and broken erythrocytes and hemosiderin deposits were the most common pigments in these macrophage [28].

The thickened trabeculae observed in the Olanzapine-treated group might allow the spleen to contract and expel the stored extra erythrocytes from the congested sinusoids. The increased thickness of trabeculae could be caused by increased fibrogenesis secondary to oxidative impairment. Lipid peroxidation might prompt and sustain an inflammatory response in which macrophages intermingle with matrix-producing cells, leading to an extension of the fibrotic tissue component. Moreover, it could induce over-expression of the key molecules in the mechanisms of fibrosis, called fibrogenic cytokines, and elevate the transcription and translation of collagen [29]. 

The alterations in T-lymphocyte levels in response to Olanzapine administration here may reflect Olanzapine-induced immunotoxicity. There was a significant reduction in the CD3 immunopositive reaction in the white pulp of the Olanzapine-treated rats. The administration of vitamin C with Olanzapine (IV) increased the CD3 immunopositive reaction. Olanzapine also induced a decrease in CD3 T-lymphocytes in the splenic periarterial lymphatic sheath. The co-administration of vitamin C treatment allowed for a renovation of these cells and a restoration of the periarterial lymphatic sheath. These were also confirmed by the morphometrical results. The current results could be due to oxidative stress induced by olanzapine. Consistent with our results, aluminum-induced oxidative stress decreased the number and density of T-lymphocytes in the spleens of pregnant rats [30]. In addition, the oxidative stress induced by monosodium glutamate, a flavor enhancer, could be responsible for the reduction in T-lymphocytes [31].

The present findings of increased TNF-α in the Olanzapine-treated group may thus appear inconsistent with previously published research. Olanzapine administration for five weeks resulted in an increase in the TNF-α levels in rat adipose tissue and the hypothalamus [32]. Ali Khalifa and Gouda suggested that anti-TNF-α was significantly increased in the spleens of rats after exposure to radiation. Moreover, the principle mechanism of apoptosis occurs via the stimulation of DNA single strand breaks and, to some extent, via triggering of TNF-α [33].

The vascular endothelial growth factor is a cytokine involved in angiogenesis and neurogenesis. It appears as a putative factor in the pathogenesis of stress-related syndromes and the effectiveness of antidepressant therapy [34]. A significant increase in the VEGF of the Olanzapine-treated group is in agreement with a previous investigation, which proved that the antidepressants increase VEGF levels in the animal models of depressive-like behavior [35]. In addition, stress elevated VEGF mRNA expression in all studied neural structures [36]. 

In the current work, the administration of vitamin C with Olanzapine ameliorated the histological changes induced by Olanzapine. This improvement included the restoration of splenic architecture, with a significant increase in CD3 and decrease in TNF-α and VEGF. In agreement, ascorbic acid is a significant key player in both the development and function of T-cells regulation [37]. Ascorbic acid is one of the most vital oxidative scavengers in extracellular fluids [12]. Using electron spin resonance spectroscopy, other investigators have studied the effects of some antipsychotics, including Olanzapine, on the formation of ROS in the whole blood of rats. Also, to test vitamin C’s protective capability, they incubated the highest concentration of Olanzapine with vitamin C. They found that Olanzapine induced the formation of ROS in the whole blood of rats, which can be decreased by the use of vitamin C [38]. The vitamin C readily scavenges ROS and RNS, such as hydroxyl, superoxide, singlet oxygen, peroxynitrite, nitroxide radicals, peroxyl, and hypochlorite [39]. Vitamin C has a number of biological activities that could possibly contribute to its immune modulating properties. It is one of the most effective antioxidants, due to its capability to readily donate electrons, therefore protecting proteins, carbohydrates, lipids, and nucleic acids from damage by oxidants produced during normal cell metabolism and/or exposures to toxins [40].

## 5. Conclusions

The current study supported the results of previous studies. It can be assumed that Olanzapine has adverse effects on spleens, as indicated by histological and immunohistochemical changes. Olanzapine exerted damage in the spleen associated with a reduction in CD3+ and increased TNF-α and VEGF. Our findings highlight the potential for the use of vitamin C as an additional therapeutic approach in the prevention of the oxidative and inflammatory effects generated by Olanzapine. Vitamin C can be used as a therapeutic agent against immune toxicity by scavenging the free radical production induced by Olanzapine. Adequate intake of vitamin C is essential for human health, as it assists immune responses and plays a role as an antioxidant. Further studies should be carried out to determine the exact mechanisms of vitamin C to improve the quality of life for patients treated with Olanzapine. 

## References

[1] Todorović N., Tomanović N., Gass P., Filipović D. (2016). Olanzapine modulation of hepatic oxidative stress and inflammation in socially isolated rats. Eur. J. Pharm. Sci..

[2] Elbakary R.A. (2017). Histological Study of the Effects of Olanzapine on the Liver of Adult Male Albino Rat with and without Vitamin, C. Egypt. J. Histol..

[3] Chawla N., Kuma S., Balhara Y.P.S. (2017). Olanzapine-induced Skin Eruptions. Indian J. Psychol. Med..

[4] Domínguez-Jiménez J.L., Puente-Gutiérrez J.J., Pelado-García E.M., Cuesta-Cubillas D., García-Moreno A.M. (2012). Liver toxicity due to olanzapine. Rev. Esp. Enferm. Dig..

[5] Houseknecht K.L., Robertson A.S., Zavadoski W., Gibbs E.M., Johnson D.E., Rollema H. (2006). Acute effects of atypical antipsychotics on whole-body insulin resistance in rats: Implications for adverse metabolic effects. Neuropsychopharmacology.

[6] Weston-Green K., Huang X.F., Deng C. (2011). Olanzapine treatment and metabolic dysfunction: A dose response study in female Sprague Dawley rats. Behav. Brain Res..

[7] Mishra A.C., Mohanty B. (2010). Effects of lactational exposure of olanzapine and risperidone on hematology and lymphoid organs histopathology: A comparative study in mice neonates. Eur. J. Pharm..

[8] Walss-Bass C., Weintraub S.T., Hatch J. (2008). Clozapine causes oxidation of proteins involved in energy metabolism: A possible mechanism for antipsychotic induced metabolic alterations. Int. J. Neuropsychopharmacol..

[9] Angelova P.R., Abramov A.Y. (2018). Role of mitochondrial ROS in the brain: From physiology to neurodegeneration. FEBS Lett..

[10] Jain S.P., Redasani V.K., Kalaskar M.G. (2011). Protective effect of Ginkgo biloba on ethanol-induced immunosuppression in rats. Eur. J. Exp. Biol..

[11] Liu J., Wang H., Zhao W. (2019). Induction of pathological changes and impaired expression of cytokines in developing female rat spleen after chronic excess fluoride exposure. Toxicol. Ind. Health.

[12] Mohamed D.S., Abdelhaliem N.G., Zakaria A.M. (2017). Histological and Immunohistochemical Study of the Possible Protective Effect of Ascorbic Acid on the Toxic Effect of Monosodium Glutamate on the Spleen of Adult Male Albino Rat. Egypt. J. Histol..

[13] Ozmen O. (2016). Endosulfan splenic pathology and amelioration by vitamin C in New Zealand rabbit. J. Immunotoxicol..

[14] Ströhle A., Wolters M., Hahn A. (2011). Micronutrients at the Interface Between Inflammation and Infection Ascorbic Acid and Calciferol. Part 1: General Overview with a Focus on Ascorbic Acid. Inflamm. Allergy Drug Targets.

[15] Wintergerst E.S., Maggini S., Hornig D.H. (2006). Immune enhancing role of Vitamin C and zinc and effect on clinical conditions. Nutr. Metab..

[16] Sang X.Z., Zheng L., Sun Q.Q., Zhang T., Li N., Cui Y., Hu R., Gao G., Cheng Z., Cheng J. (2012). The chronic spleen injury of mice following exposure to titanium dioxide nanoparticules. J. Biomed. Mater. Res..

[17] Rahman H., Aman Upaganlawar A., Upasani C. (2017). Protective Effect of Ferulic Acid Alone and in Combination with Ascorbic Acid on Aniline Induced Spleen Toxicity. Ann. Pharmacol. Pharm..

[18] Paget G.E., Barnes J.M. (1964). Evaluation of Drug Activities and Pharmacometrics.

[19] Bancroft J., Gamble M. (2008). Theory and Practice of Histological Techniques.

[20] Bancroft J.D., Gamble M. (2008). Theory and Practice of Histological Techniques.

[21] Basta-Kaim A., Kubera M., Budziszewska B., Siwanowicz J., Lasoñ W. (2002). Effect of some antipsychotic drugs on immunoreactivity in C57BL/6 mice. Pol. J. Pharm..

[22] El-Awdan S.A., Abdel-Sala O.M. (2012). Modulation of Antipsychotic-induced Oxidative Stress by Selective and Non Selective COX2 Nonsteroidal Anti-inflammatory Drugs. Pharmacologia.

[23] Victoriano M., de Beaurepaire R., Naour N., Guerre-Millo M., Quignard-Boulangé A., Huneau J.F., Mathé V., Tomé D., Hermier D. (2010). Olanzapine-induced accumulation of adipose tissue is associated with an inflammatory state. Brain Res..

[24] Eftekhari A., Azarmi Y., Parvizpur A., Eghbal M.A. (2016). Involvement of oxidative stress and mitochondrial/lysosomal cross-talk in Olanzapine cytotoxicity in freshly isolated rat hepatocytes. Xenobiotica.

[25] Kim H.K., Isaacs-Trepanier C., Elmi N., Rapoport S.I., Andreazza A.C. (2016). Mitochondrial dysfunction and lipid peroxidation in rat frontal cortex by chronic NMDA administration can be partially prevented by lithium treatment. J. Psychiatr. Res..

[26] Mazen N.F., Saleh E.Z., Mahmoud A.A., Shaalan A.A. (2017). Histological and immunohistochemical study on the potential toxicity of sliver nanoparticles on the structure of the spleen in adult male albino rats. Egypt. J. Histol..

[27] Zhang X.F., Choi Y.J., Han J.W., Kim E., Park J.H., Gurunathan S., Kim J.H. (2015). Differential nanoreprotoxicity of silver nanoparticles in male somatic cells and spermatogonial stem cells. Int. J. Nanomed..

[28] Cesta M. (2006). Normal structure, function, and histology of the spleen. Toxicol. Pathol..

[29] Poli G., Parola M. (2006). oxidative damage and fibrogenesis. Free Radic. Biol. Med..

[30] Ayuob N.N. (2013). Can vitamin E and selenium alleviate the immunologic impact of aluminium on pregnant rats’ spleens?. Cell Immunol..

[31] Hassan Z.A., Arafa M.H., Soliman W.I., Atteia H.H., Al-Saeed H.F. (2014). The Effects of Monosodium Glutamate on Thymic and Splenic Immune Functions and Role of Recovery (Biochemical and Histological study). J. Cytol. Histol..

[32] Zhang Q., He M., Deng C., Wang H., Huang X.F. (2014). Effects of olanzapine on the elevation of macrophage. infiltration and pro-inflammatory cytokine expression in female rats. J. Psychopharmacol..

[33] Ali Khalifa M.E., Gouda Z.A. (2018). The Antioxidative and Antiapoptotic Effects of Chlorophyllin on a Female Rat Spleen Exposed to Localized Gastric Radiotherapy. J. Biochem. Cell Biol..

[34] Nowacka M.M., Obuchowicz E. (2012). Vascular endothelial growth factor (VEGF) and its role in the central nervous system: A new element in the neurotrophic hypothesis of antidepressant drug action. Neuropeptides.

[35] Schmidt H.D., Duman R.S. (2007). The role of neurotrophic factors in adult hippocampal neurogenesis, antidepressant treatments and animal models of depressive-like behavior. Behav. Pharm..

[36] Nowacka-Chmielewska M.M., Paul-Samojedny M., Bielecka-Wajdman A.M., Barski J.J., Obuchowicz E. (2017). Alterations in VEGF expression induced by antidepressant drugs in female rats under chronic social stress. Exp. Ther. Med..

[37] Manning J., Mitchell B., Appadurai D.A., Shakya A., Pierce L.J., Wang H., Spangrude G.J. (2013). Vitamin C promotes maturation of T-cells. Antioxid. Redox Signal..

[38] Heiser P., Sommer O., Schmidt A.J., Clement H.W., Hoinkes A., Hopt U.T., Schulz E., Krieg J.C., Dobschütz E. (2010). Effects of antipsychotics and vitamin C on the formation of reactive oxygen species. J. Psychopharmacol..

[39] Halliwell B., Whiteman M., Packer L., Fuchs J. (1997). Antioxidant and prooxidant properties of vitamin C. Vitamin C in Health and Disease.

[40] Mandl J., Szarka A., Banhegyi G. (2009). Vitamin C: Update on physiology and pharmacology. Br. J. Pharmacol..

